# A Manual of Diseases of the Throat and Nose, Including the Pharynx, Trachea, Œsophagus, Nose and Naso-Pharynx

**Published:** 1885-03

**Authors:** W. E. Casselberry


### Book Reviews.

*A Manual of Diseases of the Throat and Nose, including the Pharynx, Larynx, Trachea, Œsophagus, Nose and Naso-Pharynx.* By Morell Mackenzie, M. D., London, Consulting Physician to the Hospital for Diseases of the Throat, etc., etc.

Volume II. *Diseases of the Œsophagus, Nose and Naso-Pharynx.* Wood's Library, August, 1884. Octavo, pp. VI., 400. New York: William Wood & Company. 1884. Chicago: W. T. Keener.

The name of Morell Mackenzie, of London, we associate intuitively, and deservedly so, with those of Turck, Czermack, Stoerk, and Schrötter, his German *confrères* and collaborators in the early development of the sciences of laryngology and rhinology. These special departments of medicine owe their very existence to the invention of various mechanical contrivances; instruments, first for precise diagnoses, then for treatment; and in perusing the pages of this work, one cannot but admire the keenness of conception and wealth of device, displayed by the author, in the many ingenious, and withal simple, inventions necessary to meet the indications.

Volume II is systematically arranged, the œsophagus, nose, and naso-pharynx, embracing sections IV., V. and VI. Each section commences with a lucid *résumé* of the anatomy of the respective organs, the clearness of which might have been still further enhanced by the insertion of a few good wood-cuts. This is followed by copiously illustrated explanations of the instruments employed, while at the head of each chapter is placed a comprehensive definition of the disease forming its subject, its synonyms, and equivalents. The history and complete bibliography of each individual affection will prove of the utmost value to future writers. They have been rendered possible only by residence of the author in the metropolis, with the most extensive medical libraries and a corps of able young assistants always at his command.

In the article upon Examination of the gullet, it is made perfectly evident that the art of auscultation of the œsophagus should no longer be regarded as an impractical refinement. Normal deglutition produces a certain sound, when of liquids and in the pharyngeal region, best described by the whispered word "*glouglou*," whilst lower down a variation, the true "œsophageal sound" becomes audible. Various diseases occasion modifications of these sounds, strongly suggestive to the familiarized ear. It is also clear from the author's experiments, that the lumen of the distended œsophagus is not circular but oval, flattened antero-posteriorly, and that œsophageal bougies should be made similar in shape, not round, but flattened antero-posteriorly, Fig. 2 representing a proper scale.

For actual visual examination of the interior of the œsophagus, Mackenzie has introduced an œsophagoscope, so simple in construction as to be available in most cases and by any physician accustomed to the use of a head reflector and to the introduction of œsophageal instruments. He has utilized it in a number of instances. On one occasion he was enabled to diagnosticate various veins within the œsophagus. His diagnosis was verified by a subsequent hæmorrhage, thus definitely located. In another patient he diagnosticated and removed a small lamella of bone—a foreign body, which was not demonstrable upon sounding with the bougie, but which was causing urgent symptoms of dysphagia.

It is to be regretted that, in this connection, the apparatus of Mikulicz designed to illuminate the interior of the œsophagus and the cardiac end of the stomach by means of the electric light, should receive but passing notice. It should have been more fully elaborated. During the winter of '81 and '82, this instrument was successfully employed by Mikulicz in Billroth's wards of the Vienna General Hospital, and especially do we recall a case in which Billroth was enabled to see a carcinoma at the cardiac end of the stomach. The apparatus is somewhat complicated, consisting, however, chiefly of a tube which carries an electric light at its distal end along the œsophagus into the stomach.

All are familiar with acute traumatic œsophagitis, resulting from irritants and caustics in process of deglutition. Perhaps the most novel of causes, which are mentioned, is the sting of insects, accidentally swallowed, which occasion, not uncommonly, a very severe form of the disease.

Acute *idiopathic* œsophagitis, on the other hand, has been so rarely recognized that it has not been mentioned in most treatises. The author's experience embraces cases of this disease, occasioned by " taking cold," extension of inflammation downward, from the pharynx, some epidemics of angina having a remarkable tendency in this direction; extension upwards of a gastritis or gastro-enteritis, the rheumatic diæthesis, and the abuse of alcohol.

Cicatricial stricture of the gullet and the various methods of treating it: gentle dilatation, forcible dilatation, internal œsophagotomy, "œsophagostomy," and "gastrostomy," are all of them comprehensively discussed. When gentle dilatation has failed, the temptation is towards internal œsophagotomy,—to cut through the stricture from within. For this purpose Mackenzie has devised an œsophagotome, not at all complicated in construction, and well adapted to its purpose. The mortality reports of this operation, however, are not encouraging, and unless one would court the immediate death of his patients he must select carefully his cases. It is obvious that where the greater portion of the tube is involved in tortuous stricture, or where the obstructing ridges are vertical in direction, it is worse than useless to go slashing around in such a locality.

Quite amusing, as well as instructive, is the relation shown to exist between spasm of the œsophagus and various psychical influences. A gentleman returned to France, after an absence of twenty years, learned that his brother's death had resulted from the bite of a dog, by which he also had been bitten. He was seized with œsophageal spasm, which ultimately proved fatal. Another patient was seized with the characteristic symptom after a dog had *tried* to bite him.

In the anatomical preface to the section upon diseases of the nose, a *fourth* turbinated body is described, present, according to Zuckerkandl, in one-third of all cases, and, therefore, to be borne in mind; and among the instruments for posterior rhinoscopy we note with pleasure the uvula holder of Voltolini figured, and the inventor's method of using it explained. We have found this to be the only instrument of the slightest value in accomplishing its purpose. It will enable one at times to obtain a good rhinoscopic image otherwise unattainable. The diagnosis between nasal polypus and hypertrophy of the turbinated bodies is specially emphasized, and with good reason. Differentiation between these two affections has not, hitherto, received attention in surgical works. Too frequently have portions of the turbinated bones been extracted by forceps under the impression that the nasal obstruction was due to polypus. "Polypi, though often bilateral, are seldom so symmetrical as is the thickening of the turbinated bodies, and whilst the color of the former is pale yellow or pink, that of the hypertrophied turbinated bodies is either bright red or dark red. Again, though the thickened mucous membrane pits a little under the probe, the entire body does not move, as in the case with polypus."

Tuberculosis of the nasal mucous membrane hitherto, apparently, a rare disease, the author believes destined to become much more common in the immediate future, because it will be looked for and will be recognized and reported. It may be seen either in the form of tumors varying from the size of a millet seed to that of a bantam's egg, or there may be but slight thickening and ulceration of the mucous membrane. Both forms show a preference for the septum, and occur usually in conjunction with tuberculosis elsewhere.

The general style of Wood's Library is well known; the print is bad, but the price is low. Other editions are published, both English and American. W. E. CASSELBERRY.

